# Effect of Caregiver’s Mental Health on Early Childhood Development across Different Rural Communities in China

**DOI:** 10.3390/ijerph15112341

**Published:** 2018-10-23

**Authors:** Siqi Zhang, Ruirui Dang, Ning Yang, Yu Bai, Lei Wang, Cody Abbey, Scott Rozelle

**Affiliations:** 1 Center for Experimental Economics of Education, Shaanxi Normal University, Xi’an 710069, Shaanxi, China; zhangsiqiceee@163.com (S.Z.); dangruiruiceee@163.com (R.D.); yangningceee@163.com (N.Y.); wangleiml@163.com (L.W.); 2 International Business School, Shaanxi Normal University, Xi’an 710069, Shaanxi, China; 3 Rural Education Action Project, Stanford University, Stanford, CA 94305, USA; cjabbey88@gmail.com (C.A); rozelle@stanford.edu (S.R.)

**Keywords:** infants and toddlers, developmental outcomes, mental health, parenting practices, rural China

## Abstract

Previous research has found that there are high rates of developmental delays among infants and toddlers in rural areas of China. Caregiver mental health problems might be one significant predictor of developmental delays among infants and toddlers, as has been found in other areas of the world. One way that the mental health of caregivers could affect early childhood development is through its effect on parenting practices. In this study, we used data from four major subpopulations of rural China to measure the correlation of caregiver mental health problems with the developmental outcomes of infants and toddlers. To do so, the study used the Bayley Scales of Infant Development III (BSID III) to examine the rates of developmental delays among 2514 rural infants/toddlers aged 6–30 months old. The results of the testing demonstrate that 48% of the sample’s infants/toddlers have cognitive delays; 52% have language delays; 53% have social-emotional delays; and 30% have motor delays. The data collection team also assessed caregiver mental health by using the Depression Anxiety Stress Scales (DASS-21) questionnaire. According to the findings, 39% of caregivers in the sample have symptoms of at least one kind of mental health problem (depression, anxiety, or stress). We also found that most caregivers do not engage in positive parenting practices, while a significant share of caregivers engage in negative parenting practices. The statistical analysis found that showing signs of mental health problems is significantly and negatively associated with infant/toddler developmental outcomes. The study also found that caregivers who show signs of mental health problems are significantly less likely to engage in interactive parenting practices. The study confirms that society needs to pay more attention to caregiver mental health problems in order to improve infant/toddler developmental outcomes in rural China and increase human capital accumulation in China as a whole.

## 1. Introduction

The existing literature has shown that human capital—the total stock of skills of a labor force—is an important driver of growth and that underinvestment in human capital can cause long-term economic problems [1]. Human capital develops at a much faster rate than conventional (nonhuman) capital, and its growth may well be the most distinctive feature of an economic system’s improvement [2,3,4]. Therefore, encouraging human capital development is one of the most basic measures that a country can take to stimulate economic growth and avoid economic stagnation. A developing economy’s underinvestment in human capital may cause it to fall into the “middle-income trap”—a state of stagnation in which a middle-income nation fails to reach high-income status [5,6]. Nations that fall into the “middle-income trap” tend to have high rates of unemployment, crime, and societal instability [7,8].

Early childhood development (ECD) is essential for human capital accumulation, and its impacts are long-lasting [9]. The first few years of life are critical, as the brain is vulnerable to biological, environmental, and psychosocial factors [10,11,12]. Heckman reported that as age increases, the return rate of human capital investment has a downward trend [13]; therefore, interventions during the first few years of life have the highest rate of return. Studies have found that the cognitive development of young children has positive effects on later life outcomes, such as educational attainment, employment, and earnings [11,14,15,16]. In contrast, infants with lower levels of cognition are more likely to result in higher unemployment rates and criminal tendencies [2,13,17].

Some studies have found that maternal mental health, especially depression, is associated with developmental delays among infants/toddlers. Infants’ early relationships with their parents (particularly the mother) lay the foundation for later personality and intellectual development [18,19,20]. Research has reported that there is a positive and significant association between maternal depressive symptoms and impaired child growth [21,22]. In developing countries, researchers found that young children of mothers who have mental health problems have lower levels of cognitive function [23]. Similarly, the problem also exists in developed countries, such as Britain and Canada [18,24].

One way that the mental health of caregivers can affect early childhood development is through affecting the ways that caregivers parent (or, in other words, their parenting practices). Research has shown that caregivers who have mental health problems do not have quality parenting practices [25]. Downey and Coyne reported that mothers who have mental health problems are more likely to interact with their children in a way that is similar to their interaction with other adults, engaging in fewer interactive parenting practices with their children, such as playing with them or singing to them [26]. Moreover, the absence of stimulating parenting practices is significantly correlated with a delay in children’s cognitive and motor development [27].

So, do developmental delays and caregiver mental health problems exist in rural communities in China? Previous research has shown that there are high rates of developmental delays among infants and toddlers in rural areas of China. To the extent that this is true, it is likely that many of these infants will not be able to fully realize their developmental potential when they are older. Two studies conducted in rural Shaanxi Province found that approximately 20% of infants—ages 6–12 months—in their samples were cognitively delayed [28,29]. Two other studies conducted in the same region found that the proportion of cognitive delay among sample toddlers (around 24–36 months) was nearly 50% [27,30]. In the most comprehensive of these studies, which used the third edition of the Bayley Scales of Infant and Toddler Development to evaluate the developmental levels of 3343 infants across four major rural subpopulations, Wang et al. found that 49% of the infant/toddler respondents were cognitively delayed; 52% suffered from language delays; 53% had social-emotional delays; and 30% had motor delays [31]. These rates are much higher than the rates of delay in normal, healthy populations, which are approximately 15% (when delays are defined as outcomes less than one standard deviation below the normalized mean [32]).

Studies also have shown that adults in China’s countryside have higher rates of mental health problems than in urban China. One study found that the depression rates among a sample of mothers and fathers of toddlers in Guangzhou were 14% and 11%, respectively [33]. In contrast, using the Zung Self-Rating Depression Scale, Wei et al. found that a much higher percentage of rural caregivers—39.8%—had depressive symptoms in poverty-stricken areas in China (in Shanxi and Guizhou Provinces) [34]. A third study comparing urban and rural individuals found that those living in rural areas had higher rates of not only major depressive disorder but also of dysthymia, somatoform disorders, adjustment disorders, and hypochondriasis than those living in urban areas [35].

While there are studies of cognitive delay among rural infants/toddlers in China, and while there are studies of caregiver mental health, there have been fewer efforts, based on large-scale data collection efforts, to document the relationship between caregiver mental health and ECD outcomes in different subpopulations across rural China. In one study, Wei et al. found that depression among caregivers was a significant predictor of toddler (aged 1–35 months) developmental delay in rural areas of Shanxi and Guizhou Province [34]. This study, however, was only conducted in remote rural mountainous areas of China. The rural landscape in China is quite diverse, both in terms of geography and level of economic development. In addition to those living in poor, remote, mountainous regions in western China and less developed plains villages in central China, many rural people now reside in migrant communities in urban areas and in large resettlement communities. To our knowledge, there have never been any studies of the correlation between caregiver mental health and developmental delays in these other types of rural communities—even though there may be significant differences in the rates and severity of the mental health of caregivers and ECD outcomes of infants/toddlers across these communities.

Previous studies of the relationship between infant/toddler developmental delays and caregiver mental health conducted in China have also rarely examined whether the pathway of the association is through the negative impact on parenting practices—although there are studies in China that document the subpar nature of parenting in many rural households. Specifically, in recent years, different research teams have shown that caregivers in rural communities rarely engage in interactive parenting practices, such as reading or singing to their child [27,31]. While some studies have concluded that one potential reason for the high prevalence of poor parenting in rural China is the absence of the understanding of what good parenting is [27,31], it is possible that poor caregiver mental health is also behind their poor parenting styles. To our knowledge, however, this question has not yet been explored by previous studies in rural China—in any of the major rural communities in China.

In this study, the overall goal is to understand the relationship between caregiver mental health and infant/toddler developmental delays across four major rural subpopulations in China: western China (mountainous) rural communities, resettlement migration communities, central China (plains) rural communities, and migrant communities. To meet this goal, we have four specific objectives. First, we report on the findings of a large-scale survey (*n* = 2514) of infant/toddler developmental outcomes across the four rural communities. Second, we describe and compare both rural caregiver mental health outcomes and parenting practices across these regions. Third, we examine the correlation between early childhood developmental outcomes and caregiver mental health. Finally, we examine the correlation between caregiver mental health and the nature of the parenting practices that are observed in the sample regions (both positive practices, such as playing with the baby, and negative practices, such as yelling at the baby).

The rest of this paper is organized as follows. The next section describes our sampling methods, data collection, and quantitative analysis strategies. The third section presents the results of our quantitative analysis. The fourth section discusses our findings and conclusions.

## 2. Methods

### 2.1. Sampling Selection

In this study, we used data from five different data collection efforts, which included survey data from four rural subpopulations: western China rural communities, resettlement communities, central China rural communities, and migrant communities. This division of China’s rural population (described below) is found in the Communiqué on Major Data of the Second National Agricultural Census of China [36]. A summary of the distribution and location of the sample households in each of these data sets can be found in Table 1.

We conducted our study in five areas of China: (A) a northwestern province, (B) a northeastern province, (C) a southwestern province, (D) a central province, and (E) a metropolis in northern China, which represent different geographic locations and different levels of economic development in China. In previous studies on the relationship between caregiver mental health and ECD outcomes among rural segments of China’s population, research was mainly concentrated in rural areas of western and central China, but due to urbanization and anti-poverty initiatives in the last few decades, the residential distribution of low-income Chinese citizens is no longer limited to the countryside. A substantial number of rural Chinese citizens now live in two kinds of communities besides traditional rural communities. First, as a result of a large flow of rural-urban migration by rural residents hoping to find higher salaries in the cities over the past few decades, migrant communities in many of China’s urban areas have formed. Second, due to poverty-relief measures, some rural residents have been relocated by the government to resettlement communities, where there is convenient transportation to county seats and urban areas, as well as fewer natural disasters than in many remote rural areas. Therefore, in this paper, we also analyze rural populations in cities (migrant communities) and resettlement migration communities, as well as western and central China rural communities. Altogether, we collected data from 2514 caregiver–child pairs across the four subpopulations. Although the overall data collection approaches of each data collection effort were similar, there were slight differences between how we conducted sampling in each kind of subpopulation. Therefore, we describe in detail how we sampled data in each of the four kinds of subpopulations below.

Our overall sampling strategy for the western China rural subpopulations is as follows. First, we randomly chose 22 nationally designated impoverished counties (the central government uses average per-capita income to designate certain counties as “impoverished” in order to focus the efforts of its poverty-reduction programs [37]) from three provinces (A, B, and C). Province A is located in northwestern China, and it is ranked 13 out of 31 in terms of provincial GDP (Gross Domestic Product) per capita (from high to low). Province B is located in northeastern China, and it is ranked 19th in terms of provincial GDP per capita. Province C, ranked 30 in terms of provincial GDP per capita, is located in southwestern China [38]. Second, we created a list of all towns in each county. After removing the county seats, we randomly selected one town in each county. Third, in each selected town, we randomly chose two or three villages for our study. Fourth, we obtained a list of all 6–24 month-old infants and toddlers (in Province A) or 6–18 month-old infants and toddlers (in Provinces B and C) in these sample villages from the local family planning officials, and all these infants and toddlers and their caregivers were enrolled in the study. In total, the western China rural communities included 2061 observations.

The sampling procedure used in the central China rural communities was similar to that used when sampling western China rural communities. We first selected one sample county in Provinces A and D, and after that, we randomly selected one town in each county. After randomly selecting the town, we then randomly selected two or three villages in each town. Once the sample villages were chosen, we obtained a list of all 6–30-month-old infants and toddlers in these sample villages, and then we chose the sampled infants and toddlers randomly. The central China rural communities included 124 observations in total.

In order to select the sample resettlement communities, we obtained a list of all resettlement communities that had over 50 6–30-month-old infants and toddlers in Provinces A and D, and then we randomly selected two or three cities in each province. After randomly selecting the cities, we randomly selected two or three resettlement communities in each city. Once the sample communities were chosen, we turned to the community family planning officials to obtain a list of all 6–30-month-old infants and toddlers in these sample communities, and then we chose the sampled infants and toddlers randomly. The resettlement communities included 129 observations in total.

In addition, we also chose sampled infants and toddlers in migrant communities of three urban areas—Metropolis E, City 1 (in Province A), and City 2 (in Province D). By referring to records held by the local family planning offices, we identified rural-urban migrant communities as well as determined which of these communities had large numbers of toddlers aged 6–30 months. Our team worked with the local governments to obtain a list of migrant communities with over 60 infants and toddlers aged 6–30 months. After randomly selecting migrant communities in each city, we obtained a list of infants and toddlers between 6 and 30 months old from the local governments of these communities and then randomly selected 200 toddlers for our sample.

In order to come up with population counts (and, hence, population shares) of the different geographical subregions, we began by matching the rural subpopulations with China’s geographical regions (we used the division of geographical regions defined by the National Bureau of Statistics of China (NBSC)). All provinces in China are divided into three regions: western, central, and eastern [39]. Specifically, we began by assuming that western China rural communities are communities in the 12 western provinces of Guangxi, Yunnan, Guizhou, Sichuan, Shaanxi, Inner Mongolia, Chongqing, Xinjiang, Tibet, Qinghai, Ningxia, and Gansu. Likewise, we assume that most of China’s central China rural communities are those in the eight provinces of central China, including Hunan, Jiangxi, Hubei, Anhui, Henan, Shanxi, Jilin, and Heilongjiang. The migrant communities are those infants and toddlers of rural families that are living in any of China’s cities. Resettlement migration communities are assumed to be in western rural communities and, as such, the total number of infants and toddlers that are in western rural communities is divided between western rural communities and resettlement migration communities.

So, where do rural individuals in China live? Although a significant portion of China’s rural infants and toddlers reside in western China rural communities (26%), the majority of them live elsewhere [30]. A considerable percentage of rural infants and toddlers also live in central China rural communities (29%) and migrant communities (13%). Taken together with infants and toddlers in resettlement villages (1.4%), over two-thirds (69%) of rural children and almost half of all of China’s youth (49% = 69% of 71% of China’s total number of children who have permanent rural residency [30]) grow up in one of these four kinds of communities (western rural communities, resettlement migration communities, central rural communities, or migrant communities).

Table 1 provides a description of the geographical locations of the sample households. There are 2514 infants and toddlers aged 6–30 months in the overall sample. The table shows that although the majority of the sample households are located in western rural communities (comprising 82% of the sample), households in the other three kinds of subpopulations still make up 18% of the sample. For the paper, we created weights based on actual population shares in order to counterbalance the disproportionate percentage of the sample located in western rural communities. We used the following formula to calculate the sampling weights: sampling weight = proportion of subpopulation in total population/proportion of subpopulation in sample. The subpopulation proportions in rural China’s total population are 37.7% for western China rural communities, 1.4% for resettlement migration communities, 42.0% for central China rural communities, and 18.8% for migrant communities. Next, in our sample, the subpopulation proportions are 82% for western rural communities, 5% for resettlement migration communities, 5% for central rural communities, and 8% for migrant communities. Therefore, the final sampling weights for each subpopulation are 0.46 for western China rural communities (equal to 37.7%/82%), 0.28 for resettlement migration villages (equal to 1.4%/5%), 8.4 for central China rural communities (equal to 42%/5%), and 2.35 for migrant communities (equal to 18.8%/8%). These weights were used to create the overall average of summary statistics, the overall average share of infant and toddler developmental outcomes, the overall average share of caregiver mental health problems, and the overall average share of parenting practices. 

### 2.2. Data Collection

We collected the data for each data set over the course of 2 years. Data set 1 was collected in the winter of 2015–2016; data set 2 was collected in the autumn of 2015; all other data sets were collected in the spring of 2017. For each data set, we recruited local college students to serve as enumerators. Before heading to the target site to collect data, we conducted a 15-day training program for the enumerators.

#### 2.2.1. Data Description

We collected five types of information from each caregiver–child pair: early childhood development data, information about the caregiver’s mental health, whether or not caregivers engage in interactive parenting practices (the variable takes on the value of 1 if caregivers engage in the respective parenting practice, and 0 if otherwise), toddler characteristics (like the toddler’s age in months, gender, and whether or not they were born prematurely), and household characteristics. In this section, we describe these five types of information in detail.

#### 2.2.2. Assessing Early Childhood Development

In order to assess early childhood development, we administered the Third Edition of the Bayley Scales of Infant and Toddler Development (BSID-III), which is a multidimensional diagnostic tool for assessing ECD that is widely used around the world [40]. The BSID-III assesses ECD across five dimensions or standardized scales, four of which we used to measure the development of the infants in our sample: the cognitive scale, which measures information processing skills, such as concept formation and memory; the language scale, which measures both receptive and expressive communication skills; the social-emotional scale, which measures functional emotional skills, including internal emotional regulation and social responsiveness; and the motor scale, which measures fine and gross motor skills. The cognitive, language, and motor scales evaluate the infant based on his/her performance on a number of tasks, whereas the social-emotional scale depends on the responses of the infant’s caregiver to a series of questions. The four scales conduct such measurements taking into account both the infant’s gestational and chronological ages. Previous research has demonstrated that the four scales of the BSID-III have high inter- and intra-rater reliability agreement, high internal consistency, and high test-retest stability, regardless of which cultural context they are tested [40,41,42,43,44].

According to BSID-III guidelines, we transformed the raw scores into composite scores [32]. We also examined the prevalence rate of developmental delays for the entire sample and for each rural subpopulation as well. A delay in any of the four measures of development is defined as a score at least one standard deviation below the mean. We compared the scores of the infants and toddlers in our sample to the mean and standard deviation of documented distributions of BSID scores in reference populations. The mean score (SD) is different for each of the four indices: 105 (9.6) for the cognitive scale [45,46], 109 (12.3) for the language scale [46], 100 (15) for the social-emotional scale [32], and 107 (14) for the motor scale [45,47].

#### 2.2.3. Assessing Caregiver’s Mental Health

All households were administered the Depression Anxiety and Stress Scale-21 (henceforth, DASS-21), a shortened version of the DASS-42 scales, developed by Lovibond and Lovibond [48]. The DASS-21 is a self-report questionnaire consisting of 21 items measuring distress levels over the previous week along three dimensions—depression, anxiety, and stress. The DASS-21 cannot be interpreted as a tool for direct clinical diagnosis [48], but it is designed to be a quantitative measure of the severity of depression, anxiety, and stress symptoms. Henry and Crawford found both adequate construct validity and high reliabilities of the DASS-21. In addition, the authors established that the DASS-21 scales “contain variance that is specific to each scale” (p. 238), while also indexing “a substantial common factor (e.g., general psychological distress)” (p. 238) [49]. Chan et al. established the cross-cultural validity of the DASS-42 in China. Several years later, Wang et al. similarly established the validity for the DASS-21, also in China [50,51]. The DASS cut-off scores for identifying caregiver symptoms used in our study are the same as the cutoff scores cited in the above studies.

Scaled scores for the DASS-21 subscales of depression, anxiety, and stress were derived by totaling the scores for each subscale and multiplying by 2. The participants were divided into the following categories: normal (0–9 for depression, 0–7 for anxiety, and 0–14 for stress), mild (10–13 for depression, 8–9 for anxiety, and 15–18 for stress), moderate (14–20 for depression, 10–14 for anxiety, and 19–25 for stress), severe (21–27 for depression, 15–19 for anxiety, and 26–33 for stress), and extremely severe (≥28 for depression, ≥20 for anxiety, and ≥34 for stress). A score of DASS ≥ 10 was used to identify caregivers who have symptoms of depression, a score of DASS ≥ 8 was used to identify caregivers who have symptoms of anxiety, and a score of DASS ≥ 15 was used to identify caregivers who have symptoms of stress.

#### 2.2.4. Interactive Parenting Practices 

To assess caregiver parenting practices—both positive and negative—we asked the primary caregivers whether or not they had engaged in a number of interactive practices the previous day: told stories to the baby, read books to the baby, sang song to the baby, used toys to play with the baby, and the number of times the caregiver expressed affection to the baby. We also asked them whether or not the household has two or more children’s books [52,53,54]. In addition, we asked the primary caregivers how often they engaged in the following negative parenting practices: raise voice or yell at baby, spank the baby, take away toys from the baby, or do not explain to the baby why his or her behavior is inappropriate [55,56]. Each respective variable took a value of 1 if the caregiver answered yes and 0 if the caregiver answered no.

#### 2.2.5. Child and Household Characteristics

Besides data on developmental status, we also recorded basic background information about the infants and toddlers. This included the infant and toddler age in months, gender, and whether or not he or she was born prematurely. The age of the toddler was taken from each child’s birth certificate.

In addition, we collected information on each household. This included the identity of the primary caregiver (e.g., mother or grandmother), the education level of the mother, the education level of the father, whether the father and mother lived at home with the child (or was living out of the village as a migrant worker), and a variable measuring whether or not the household was receiving social security support (Dibao). The survey team also collected data to measure family assets. We asked caregivers whether or not their households had the following items in order to construct polychoric principal components and develop a family asset index: tap water, toilet, water heater, washing machine, computer, internet, refrigerator, air conditioner, motor or electronic bicycle, and car.

### 2.3. Statistical Analysis

In order to measure the correlation between caregiver’s mental health and early childhood developmental outcomes, we constructed a model as follows:

Developmental Outcomes _i_ = β _0_ + β _1_ mental health _i_ + X _i_ θ + u _i_ (1)

where the dependent variable, Developmental Outcomes _i_, indicates the standardized Bayley test scores (cognitive, language, social-emotional, or motor scores) of the infant i. All of these scores are continuous variables. The variable mental health _i_ represents the score of the mental health scale of the caregiver of infant i. We ran regressions using both continuous variables and dummy variables. The continuous variables include depression scores, anxiety scores, and stress scores. The dummy variables include whether the caregiver has moderate depression symptoms whether the caregiver has moderate anxiety symptoms, and whether the caregiver has moderate stress symptoms. The term X _i_ is a vector of covariates that are included to capture characteristics of the children and their household, including the child’s gender, age, prematurity, birth status, whether the mother was the primary caregiver, maternal age, maternal educational level, the father’s educational level, whether the mother was living at home, whether the father was living at home, whether the household received social security support, and the family asset index. All these covariates are dummy variables. We accounted for the clustered at the village level and controlled for Bayley tester fixed effects. We also controlled for county fixed effects.

We also created a model to measure the correlation between the caregiver’s mental health and the interactive parenting practices. The model is as follows:

Parenting Practices _i_ = β _0_ +β _1_ mental health _i_ +X _i_ θ + u _i_ (2)

where the dependent variable, Parenting Practices _i_, indicates the positive and negative parenting practices of the infant i. The other variables are the same as those in the Development Outcome model above.

The child and household characteristics, childhood developmental outcomes, and caregiver mental health were analyzed by using the mean and standard deviation. All correlational analyses were performed using STATA 14.2 (Stata 14.2 Statistical Software, Stata Corp LLC, College Station, TX, USA). *p*-values below 0.05 were considered statistically significant.

## 3. Results

This section presents our descriptive and correlational analysis of the relationship between the caregiver’s mental health and early childhood developmental outcomes across four major rural subpopulations in China: western China rural communities, resettlement migration communities, central China rural communities, and rural-urban migrant communities. For comparisons across subpopulations, we used western China as the standard for comparison because the majority of existing research on early childhood development in western China has been conducted in poor, mountainous regions in western China.

The structure of this section of the paper is as follows. First, we present descriptive statistics that describe the child characteristics and household characteristics of the sample. Second, we describe the rates of child developmental delays, the prevalence of caregiver mental health symptoms, and the shares of caregivers that engage in interactive parenting practices in our sample (both overall and by the different rural subpopulations). Third, we measure the association between caregiver mental health and child development outcomes using OLS (ordinary least squares) analysis across these populations. Finally, we examine the association between caregiver’s mental health and parenting practices using OLS analysis across these subpopulations.

### 3.1. Child and Household Characteristics

Table 2 shows the basic socioeconomic and demographic characteristics of these four major subpopulations. In the overall sample, there are 2514 observations. When looking at the characteristics of the infants/toddlers in the sample, the data show that the average age of the infants/toddlers was 16.04 months. Over half (53%) were male. Around 6% of sample children were born prematurely. When compared to national data from across rural China, the share of males and the share of those that were born prematurely in our data set are close to such measures in China’s national statistical databases (National Bureau of Statistical of China, 2017; URL: http://www.stats.gov.cn/tjsj/ndsj/2017/indexeh.htm).

When looking at household characteristics, the mother was the primary caregiver in 68% of households; in most of the other households, the grandmother was the primary caregiver. About 69% of mothers were older than 25 years, and 34% of the mothers had completed more than 9 years of schooling. About 34% of fathers had completed more than 9 years of schooling. In 80% of the cases, the mother stayed at home, whereas about 49% of fathers stayed at home. Around 11% of sample families received the Minimum Living Standard Guarantee Payments, a form of government welfare for the lowest-income families nationwide.

We can see that parents in migrant communities had the highest levels of educational attainment. Sixty-one percent (61%) of mothers from migrant communities had attained at least 9 years of education, compared to only 22% of those in western China rural communities, 36% of those in resettlement villages, and 32% of those in central China rural communities. Similarly, the father’s educational level in migrant communities (54%) was significantly greater than those in western China rural communities (25%), resettlement migration communities (37%), and central China rural communities (32%).

From these data, we also found differences regarding the share of mothers and fathers who stayed at home between the different subsamples, finding that significantly more mothers lived at home in migrant communities (92%) than in other communities (80% or fewer). Similarly, a significantly greater percentage of fathers in migrant communities (70%) stayed at home as well. If a parent stayed at home, this meant that the parent was living with the child (and did not reside outside of the household for extended periods of time).

Besides having overall higher education levels and higher rates of living at home with their infants and toddlers, we found that migrant communities also had better economic environments than other communities. For example, we can see the family assets index in migrant communities (1.14) was significantly greater than that in the other subpopulations (−0.15 for western rural communities; 0.27 for resettlement migration communities; and 0.37 for central rural communities). Out of all the subpopulations, migrant communities also had the lowest share of sampled residents (9%) receiving Social Security (Dibao) payments (an indicator of low income).

### 3.2. Comparing Early Childhood Developmental Outcomes, Prevalence of Caregiver Mental Health Problems, and Parenting Practices across Subpopulations

The early childhood developmental outcomes from the 2514 toddlers in the sample are shown in Table 3. The definition of delay in cognitive, language, social-emotional, or motor development is at least one standard deviation lower than the mean on each of the respective scales. In the total sample, we found that more than half of sample children were suffering from delays in cognitive, language, and social-emotional development (48%, 52%, and 53%). About 30% of sample children were delayed in motor development.

When comparing developmental delays across subpopulations, the rates of delay in the development of cognitive (42%), language (40%), social-emotional (39%), and motor skills (18%) among migrant children were lower than those in the other subpopulations (those for cognitive delays are 47–53%; those for language delays are 50–61%; those for social-emotional delays are 53–58%; and those for motor delays are 30–36%).

The data suggest that a large share of caregivers were depressed, anxious, or stressed on the DASS-21 scale (Table 3). Of the full sample, 25% of caregivers were depressed, 29% were anxious, and 16% were stressed. The high prevalence of caregiver’s mental health issues is worrisome when considering that caregivers might have symptoms of more than one kind of mental disorder at the same time. Indeed, we can see from the data that 39% of caregivers showed signs of more than one kind of mental health problem.

When comparing the four subpopulations, we found that there was little difference between them in terms of the prevalence of caregiver mental health problems. Depending on the subpopulation, between 23% and 28% of caregivers showed signs of depression, between 21% and 33% of caregivers showed signs of anxiety, and between 15% and 21% of caregivers showed signs of stress. There were no significant differences between the subpopulations in regard to the prevalence of any of these mental health issues. Overall, between 37% and 43% of caregivers had symptoms of more than one kind of mental health problem, depending on the subpopulation in question.

Finally, we compared positive and negative caregiver parenting practices across the four populations (Table 4). In terms of the full sample, we found that less than one-fifth of the caregivers in these subpopulations engaged in positive parenting practices, such as storytelling (17%) and book reading (8%). Less than half of the caregivers sang songs to their children (35%) and had over two children books in the household (38%). Caregivers in western rural communities were much less likely to engage in most of these behaviors than those in central China rural communities and, in particular, migrant communities. In fact, for all six of the variables related to positive parenting practices, the percentage of rural-urban migrant caregivers who engaged in these behaviors was higher (in several cases, by more than 10 percentage points). We also found that over a third of rural caregivers engaged in negative parenting practices, such as raising one’s voice or yelling at the baby (53%), spanking the baby (42%), taking away toys from the baby (34%), and neglecting to explain why the baby’s behavior is inappropriate (33%). For negative parenting behaviors, there was almost no significant difference between the subpopulations. 

### 3.3. Correlations between Caregiver Mental Health and Early Childhood Developmental Outcomes

Table 5 and Table 6 show the correlations between caregiver mental health and early childhood developmental outcomes after adjusting for potential confounders. According to the findings, all three of the mental health disorders were significantly correlated with early childhood developmental outcomes. In Table 5, we find that the caregiver depression score had a significantly negative association with the social-emotional scores of the study’s infants and toddlers. Similarly, the caregiver anxiety scores had a significantly negative association with infant/toddler cognitive, language, and social-emotional scores. The caregiver stress scores also had a significantly negative association with the language scores of infants and toddlers in the sample.

Table 6 demonstrates that when caregivers had symptoms of depression or anxiety of at least moderate intensity according to their scores on the DASS-21, their infants and toddlers had significantly lower social-emotional scores than others. The data also tell us that the infants and toddlers of caregivers who had symptoms of at least moderate or severe anxiety had significantly lower language scores.

### 3.4. Correlates between Caregiver’s Mental Health and Parenting Practices

The previous subsection shows that, overall, the mental health of caregivers is significantly related to parenting practices: the infants and toddlers of caregivers who show signs of mental health conditions like depression, anxiety, or stress are more likely to have poor developmental outcomes. In this section, we examine whether or not parenting practices—which previous research has shown to be closely linked with developmental outcomes—are also associated with caregiver mental health problems [27,34]. If we find that they are, then it is likely that subpar parenting practices can serve as at least one reason why poor mental health is related to infant/toddler developmental delays.

From Table 7 and Table 8, it can be seen that caregivers who had at least moderate or higher severity symptoms related to the DASS-21 depression, anxiety, or stress scales were less likely to engage in positive parenting practices (such as telling their baby a story, reading their baby a book, singing their baby a song, or playing games with or expressing affection to their baby). Stress had by far the broadest correlation with parenting practices, as caregivers with stress were significantly less likely to implement positive parenting practices across all of the four indices.

Conversely, caregivers who had at least moderate and higher severity symptoms on the depression, anxiety, or stress scores of the DASS-21 were more likely to engage in negative parenting practices (such as raising their voice or yelling at their baby, spanking the baby, taking away toys from the baby, and not explaining to the baby why his or her behavior is inappropriate). In this case, depression and anxiety seemed to show the strongest correlation, having positive associations with three of the four indices.

## 4. Discussion

This study examined data from 2514 rural infants/toddlers aged 6–30 months old in four major rural subpopulations in China to better understand the relationship between caregiver mental health and early childhood developmental delays in rural China. We began our analysis with four specific objectives: to describe infant/toddler developmental outcomes across the four rural communities; to explore the prevalence of caregiver mental health problems and the share of caregivers who engage in (positive and negative) parenting practices; to examine the correlation between caregiver mental health and childhood development; and to examine the correlation between caregiver mental health and parenting practices.

Ultimately, we found that, regardless of the subpopulation, there was a high prevalence of developmental delays among the study’s infants and toddlers (including delays in cognition, language, social-emotional development, and motor skills). Overall, 48% of the sample suffered from cognitive delays, 52% suffered from language delays, 53% suffered from social-emotional delays, and 30% suffered from motor delays. These rates of delay are several times higher than that in a healthy population (9.1–13.8%) [57]. We also found that there was little variation between the developmental outcomes of different subpopulations. The one exception was rural-urban migrant communities, which had significantly lower rates of delay relative to the other subpopulations (although they were still much higher than those in a healthy population). Our results regarding developmental outcomes are consistent with the results of a previous study by Luo et al., who also found that around 50% of the sample suffered from cognitive and language delays, and 35% of the sample suffered from social-emotional delays [53].

Just as we found that developmental delays among toddlers were highly prevalent, we also discovered that mental health problems were widespread among their caregivers: 39% of them had symptoms of at least one kind of mental health problem (depression, anxiety, and stress). Rates of depression were between 23% and 28% across the four subpopulations—much higher than the results of a previous study conducted in rural areas of Liaoning Province, which is located in northeastern China (5.9% [58]). The rates of anxiety in our study were between 21% and 33%, which also are much higher than the results of previous research conducted in rural areas of Shandong Province (12.2%) [59]. Finally, rates of stress ranged from 15% to 21%. Ultimately, mental health issues were a serious problem across all of the communities: none of the rural subpopulations experienced significantly higher rates of mental health issues than the others. Clearly, depression, anxiety, and stress as measured by the DASS-21 scale are a serious problem in rural China.

We also showed that the quality of interactive parenting in rural China is poor. Positive parenting practices in these communities were rare. Overall, less than one-fifth of the caregivers engaged in storytelling, book reading, and expressing affection to the baby, while less than half sang songs to their toddlers and had over two children books at home. In contrast, negative parenting practices were alarmingly common: over one-third of caregivers raised their voice or yelled at the baby, spanked the baby, took away toys from the baby, and neglected to explain why the baby’s behavior is inappropriate.

The analysis demonstrated both poor ECD outcomes (that is, high rates of delay) and parenting practices (both positive and negative) were closely linked with the caregiver mental health outcomes. Caregivers who showed symptoms of moderate or severe depression or anxiety were significantly more likely to have infants and toddlers with social-emotional delays. Anxiety was also correlated with lower cognitive and language scores among the infants and toddlers. Our findings thus support the results of previous research that identify links between maternal depression/anxiety and early developmental outcomes among infants/toddlers in poor rural China [34] and other developing countries [60,61,62,63,64,65]. Perhaps most noteworthy, the analysis discovered that caregiver mental health issues were significantly correlated to several kinds of parenting behaviors (both positive and negative). Our study reflects the results of numerous studies in other countries that show that mental health problems can have adverse effects on parenting practices, causing parents to have fewer positive interactions with their infants and toddlers, to have higher rates of negative interactions and hostility, to communicate less effectively, and to be less responsive to child behavior [25,26,66,67,68,69]. Overall, for positive parenting practices, such as singing songs or expressing affection to the baby, stress was the most broadly correlated: caregivers who showed signs of moderate or severe stress were significantly less likely than those who did not show such signs to engage in four out of the six of these practices. On the other hand, for negative parenting practices, such as yelling at the baby or spanking the baby, depression and anxiety appeared to have the broadest correlations: caregivers with either of these mental health symptoms were significantly more likely to engage in three out of four of these behaviors. To our knowledge, although the international literature discusses parenting practices as one of the pathways between poor caregiver mental health and poor infant/toddler developmental outcomes [60,70], no large-scale studies conducted in rural China have ever demonstrated this.

Our findings thus suggest that a major reason why conditions like depression and anxiety seem to affect early childhood development outcomes is that these conditions influence the way parents interact with their children. In other words, caregivers who are depressed, anxious, or stressed are less likely to interact with their infant/toddler in ways that are helpful for their development, and they are more likely to interact with their infants/toddlers in ways that are harmful to their development.

It is important to note, however, that caregiver mental health is only one of many factors that may influence parenting practices and, ultimately, ECD outcomes, which is apparent in the results of our study. Despite the high prevalence of depression, anxiety, and stress symptoms in migrant communities being similar to other kinds of communities, significantly more caregivers in this subpopulation engaged in positive parenting practices, and ECD outcomes in these migrant communities were overall superior to those in other kinds of communities. This suggests that other factors (such as maternal education level, which in our sample was by far the highest in migrant communities) can also influence the quality of parenting.

## 5. Conclusions

This study has several strengths, as well as some limitations. In terms of strengths, this is the first study conducted in rural China to analyze the connection not only between caregiver mental health and ECD outcomes but also between caregiver mental health and a possible intermediary variable: positive and negative parenting practices. In addition, this is also the first study using the Bayley Scales of Infant Development III and the Depression Anxiety Stress Scales (DASS-21) to analyze ECD outcomes and their connection to caregiver mental health across four different rural subpopulations in China.

Like any study, this one also has several limitations. First, because of the cross-sectional nature of our study, we were not able to draw causal conclusions between infant/toddler developmental delays and the two sets of outcome variables (caregiver mental health/parenting practices). Second, as we used the parent-reported social-emotional scale to screen for infant/toddler social-emotional developmental delays, it is possible that some caregivers—especially those with mental health issues—did not accurately report the social-emotional development of their infants/toddlers. Third, although our data set is large and covers four major rural subpopulations in China, our sample did not include rural households in eastern China nor families who live in county seats and towns. Therefore, there is a need for further research to be conducted in these areas of rural China.

In summary, our analysis suggests that there are high rates of developmental delays (cognitive, language, social-emotional, and motor skills) among infants and toddlers aged 6–30 months across four major rural subpopulations in China and that high rates of mental health disorders among caregivers are significantly correlated with a number of these poor ECD outcomes. Although our findings are not causal, we found evidence that one mechanism by which caregiver mental health might be affecting ECD outcomes is potentially through influencing parenting practices, decreasing the rate of positive parenting practices and increasing the rate of negative parenting practices. The implications of this study are that academic researchers, policymakers, and Chinese society overall need to not only pay more attention to early childhood development in rural areas but also to address one of the potential root causes of the problem: caregiver mental health issues and poor parenting practices.

## Figures and Tables

**Table 1:** Summary of the distribution and location of data sets 1–5.

Dataset	Location of Study	Date	Community Type	Ages of Children	Number of Observations
1	Province A	2015–2016	Western China Rural Communities	6–24 months	1712
2	Provinces B and C	2015	Western China Rural Communities	6–18 months	349
3	Provinces A and D	2017	Resettlement Migration Communities	6–30 months	129
4	Provinces A and D	2017	Central China Rural Communities	6–30 months	124
5	City 1 (in Province A); City 2 (in Province D); Metropolis E	2017	Migrant Communities	6–30 months	200

**Table 2:** Summary statistics.

Variables	Full Sample	Western China Rural Communities	Resettlement Communities	Central China Rural Communities	Migrant Communities	Difference: (2)−(3)	Difference: (2)–(4)	Difference: (2)–(5)
	Mean	Mean	Mean	Mean	Mean	*p*-value	*p*-value	*p*-value
	(SD)	(SD)	(SD)	(SD)	(SD)			
	(1)	(2)	(3)	(4)	(5)	(6)	(7)	(8)
Child characteristics								
Age	16.04	14.57	16.50	17.42	15.90	<0.01	<0.01	<0.01
(in months)	(6.53)	(5.09)	(6.85)	(7.32)	(6.64)			
Male	0.53	0.51	0.55	0.57	0.47	0.37	0.17	0.35
(1 = yes)	(0.50)	(0.50)	(0.50)	(0.50)	(0.50)			
Premature	0.06	0.05	0.03	0.06	0.10	0.40	0.38	<0.01
(1 = yes)	(0.24)	(0.21)	(0.17)	(0.25)	(0.29)			
Household characteristics								
Primary caregiver	0.68	0.74	0.58	0.60	0.73	<0.01	<0.01	0.93
(1 = mother)	(0.47)	(0.44)	(0.50)	(0.49)	(0.44)			
Maternal age	0.69	0.65	0.71	0.69	0.74	0.23	0.36	<0.01
(1 = above 25 years)	(0.46)	(0.48)	(0.46)	(0.46)	(0.44)			
Maternal education level	0.34	0.22	0.36	0.32	0.61	<0.01	<0.01	<0.01
(1 = 9 years or higher)	(0.47)	(0.41)	(0.48)	(0.47)	(0.49)			
Father education level	0.34	0.25	0.37	0.32	0.54	<0.01	0.09	<0.01
(1 = 9 years or higher)	(0.47)	(0.44)	(0.49)	(0.47)	(0.50)			
Mother stays at home	0.80	0.80	0.74	0.76	0.92	0.09	0.28	<0.01
(1 = yes)	(0.40)	(0.40)	(0.44)	(0.43)	(0.27)			
Father stays at home	0.49	0.48	0.30	0.40	0.70	<0.01	0.10	<0.01
(1 = yes)	(0.50)	(0.50)	(0.46)	(0.49)	(0.46)			
Family asset index	0.32	−0.15	0.27	0.37	1.14	<0.01	<0.01	<0.01
	(1.19)	(1.20)	(1.22)	(1.08)	(0.88)			
Household receives socialsecurity support (1 = yes)	0.11	0.11	0.10	0.12	0.09	0.79	0.66	0.43
	(0.31)	(0.31)	(0.30)	(0.33)	(0.29)			
Observations	2514	2061	129	124	200			

Notes: We used sampling weights to calculate the summary statistics for each observation. We used the following formula to calculate the sampling weights: sampling weight = proportion of subpopulation in total population/proportion of subpopulation in sample. The subpopulation proportions in rural China’s total population are 37.7% for western China rural communities, 1.4% for resettlement migration communities, 42.0% for central China rural communities, and 18.8% for migrant communities. Next, in our sample, the subpopulation proportions are 82% for western rural communities, 5% for resettlement migration communities, 5% for central rural communities, and 8% for migrant communities. Therefore, the final sampling weights for each subpopulation are 0.46 for western China rural communities (equal to 37.7%/82%), 0.28 for resettlement migration villages (equal to 1.4%/5%), 8.4 for central China rural communities (equal to 42%/5%), and 2.35 for migrant communities (equal to 18.8%/8%). For the child and household characteristics, the first column shows the mean and standard deviation of each characteristic for the full sample; column 2 shows statistics for children and households in western China rural communities; column 3 shows statistics for children and households in resettlement communities; column 4 shows statistics for children and households in central China rural communities; column 5 shows statistics for children and households in migration communities. Column 6 shows the *p*-value of the difference between column 2 and column 3; column 7 shows the *p*-value of the difference between column 2 and column 4; column 8 shows the *p*-value of the difference between column 2 and column 5. We asked caregivers whether or not their households had the following items in order to construct polychoric principal components and develop the family asset index: tap water, toilet, water heater, washing machine, computer, internet, refrigerator, air conditioner, motor or electronic bicycle, and car.

**Table 3:** Child development outcomes and caregiver’s mental health across different subpopulations.

Variables	Full Sample	Western China Rural Communities	Resettlement Communities	Central China Rural Communities	Migrant Communities	Difference: (2)–(3)	Difference: (2)–(4)	Difference: (2)–(5)
	Mean	Mean	Mean	Mean	Mean	*p*-Value	*p*-Value	*p*-Value
	(SD)	(SD)	(SD)	(SD)	(SD)			
	(1)	(2)	(3)	(4)	(5)	(6)	(7)	(8)
Child development outcomes								
Cognitive delay	0.48	0.53	0.50	0.47	0.42	0.55	0.17	<0.01
(1 = yes)	(0.50)	(0.50)	(0.50)	(0.50)	(0.49)			
Language delay	0.52	0.61	0.53	0.50	0.40	0.10	0.02	<0.01
(1 = yes)	(0.50)	(0.49)	(0.50)	(0.50)	(0.49)			
Social-emotional delay	0.53	0.58	0.65	0.53	0.39	0.12	0.27	<0.01
(1 = yes)	(0.50)	(0.49)	(0.48)	(0.50)	(0.49)			
Motor delay	0.30	0.36	0.33	0.30	0.18	0.57	0.18	<0.01
(1 = yes)	(0.46)	(0.48)	(0.47)	(0.46)	(0.39)			
Caregiver’s mental health								
Depression	0.25	0.23	0.26	0.28	0.23	0.48	0.17	0.90
(1 = yes)	(0.43)	(0.42)	(0.44)	(0.45)	(0.42)			
Anxiety	0.29	0.28	0.33	0.32	0.21	0.27	0.31	0.05
(1 = yes)	(0.45)	(0.45)	(0.47)	(0.47)	(0.41)			
Stress	0.16	0.15	0.21	0.15	0.20	0.06	0.86	0.07
(1 = yes)	(0.37)	(0.35)	(0.41)	(0.36)	(0.40)			
Depression/Anxiety/Stress	0.39	0.37	0.43	0.43	0.38	0.17	0.17	0.81
(1 = yes)	(0.49)	(0.48)	(0.50)	(0.50)	(0.49)			
Observations	2514	2061	129	124	200			

Notes: We used sampling weights to calculate the summary statistics for each observation. We used the following formula to calculate the sampling weights: sampling weight = proportion of subpopulation in total population/proportion of subpopulation in sample. The subpopulation proportions in rural China’s total population are 37.7% for western China rural communities, 1.4% for resettlement migration communities, 42.0% for central China rural communities, and 18.8% for migrant communities. Next, in our sample, the subpopulation proportions are 82% for western rural communities, 5% for resettlement migration communities, 5% for central rural communities, and 8% for migrant communities. Therefore, the final sampling weights for each subpopulation are 0.46 for western China rural communities (equal to 37.7%/82%), 0.28 for resettlement migration villages (equal to 1.4%/5%), 8.4 for central China rural communities (equal to 42%/5%), and 2.35 for migrant communities (equal to 18.8%/8%). For the descriptive statistics of child development outcomes and the caregiver’s mental health, column 6 shows the *p*-value of the difference between column 2 and column 3; column 7 shows the *p*-value of the difference between column 2 and column 4; column 8 shows the *p*-value of the difference between column 2 and column 5.

**Table 4:** Parenting practices across different subpopulations.

Variables	Full Sample	Western China Rural Communities	Resettlement Communities	Central China Rural Communities	Migrant Communities	Difference: (2)–(3)	Difference: (2)–(4)	Difference: (2)–(5)
	Mean	Mean	Mean	Mean	Mean	*p*-Value	*p*-Value	*p*-Value
	(SD)	(SD)	(SD)	(SD)	(SD)			
	(1)	(2)	(3)	(4)	(5)	(6)	(7)	(8)
Positive Parenting Practices								
Told story yesterday	0.17	0.09	0.15	0.19	0.28	0.05	<0.01	<0.01
(1 = yes)	(0.37)	(0.29)	(0.36)	(0.39)	(0.45)			
Read book yesterday	0.08	0.03	0.05	0.07	0.20	0.20	0.02	<0.01
(1 = yes)	(0.27)	(0.18)	(0.23)	(0.26)	(0.40)			
Sang song yesterday	0.35	0.28	0.29	0.35	0.52	0.77	0.05	<0.01
(1 = yes)	(0.48)	(0.45)	(0.45)	(0.48)	(0.50)			
Played game yesterday	0.61	0.52	0.56	0.66	0.69	0.37	<0.01	<0.01
(1 = yes)	(0.49)	(0.50)	(0.50)	(0.48)	(0.46)			
Over two child books in household	0.38	0.26	0.33	0.41	0.55	0.08	<0.01	<0.01
(1 = yes)	(0.49)	(0.44)	(0.47)	(0.49)	(0.50)			
Times expressed affection to baby	13.02	8.35	15.50	15.02	17.82	<0.01	<0.01	<0.01
(times)	(15.25)	(7.65)	(21.58)	(17.32)	(18.63)			
Negative Parenting Practices								
Sometimes raise voice or yell at baby	0.53	0.51	0.53	0.56	0.49	0.70	0.23	0.70
(1 = yes)	(0.50)	(0.50)	(0.50)	(0.50)	(0.50)			
Sometimes spank the baby	0.42	0.36	0.43	0.51	0.33	0.10	<0.01	0.29
(1 = yes)	(0.49)	(0.48)	(0.50)	(0.50)	(0.47)			
Sometimes take away toys from baby(1 = yes)	0.34	0.29	0.32	0.40	0.32	0.53	0.01	0.40
	(0.47)	(0.45)	(0.47)	(0.49)	(0.47)			
Sometimes do not explain why baby’s behavior is not appropriate to him/her (1 = yes)	0.33	0.33	0.36	0.35	0.28	0.54	0.71	0.15
	(0.47)	(0.47)	(0.48)	(0.48)	(0.45)			
Observations	2514	2061	129	124	200			

Notes: We used sampling weights to calculate the summary statistics for each observation. We used the following formula to calculate the sampling weights: sampling weight = proportion of subpopulation in total population/proportion of subpopulation in sample. The subpopulation proportions in rural China’s total population are 37.7% for western China rural communities, 1.4% for resettlement migration communities, 42.0% for central China rural communities, and 18.8% for migrant communities. Next, in our sample, the subpopulation proportions are 82% for western rural communities, 5% for resettlement migration communities, 5% for central rural communities, and 8% for migrant communities. Therefore, the final sampling weights for each subpopulation are 0.46 for western China rural communities (equal to 37.7%/82%), 0.28 for resettlement migration villages (equal to 1.4%/5%), 8.4 for central China rural communities (equal to 42%/5%), and 2.35 for migrant communities (equal to 18.8%/8%). For the parenting practices, the first column shows the mean and standard deviation for the full sample; column 2 shows statistics for parenting practices in western China rural communities; column 3 shows statistics for parenting practices in resettlement communities; column 4 shows statistics for parenting practices in central China rural communities; column 5 shows statistics for parenting practices in migration communities. Column 6 shows the *p*-value of the difference between column 2 and column 3; column 7 shows the *p*-value of the difference between column 2 and column 4; column 8 shows the *p*-value of the difference between column 2 and column 5.

**Table 5:** Correlations between caregiver’s mental health and child development outcomes using ordinary least squares, *n* = 2514.

Variables		Cognitive Scores	Language Scores	Social-Emotional Scores	Motor Scores
		(1)	(2)	(3)	(4)
(1)	Depression Scores	0.01	−0.01	−0.02 ***	−0.00
		(0.01)	(0.00)	(0.00)	(0.01)
	Controls	YES	YES	YES	YES
	Tester Fixed Effects	YES	YES	YES	YES
	Adjusted R-squared)	0.25	0.27	0.21	0.19
(2)	Anxiety Scores	−0.01 *	−0.02 ***	−0.02 ***	−0.01
		(0.01)	(0.01)	(0.01)	(0.01)
	Controls	YES	YES	YES	YES
	Tester Fixed Effects	YES	YES	YES	YES
	Adjusted R-squared)	0.25	0.27	0.21	0.19
(3)	Stress Scores	−0.00	−0.01 **	−0.01	−0.00
		(0.00)	(0.00)	(0.00)	(0.00)
	Controls	YES	YES	YES	YES
	Tester Fixed Effects	YES	YES	YES	YES
	Adjusted R-squared)	0.25	0.27	0.20	0.19

Notes: We used sampling weights to calculate the summary statistics for each observation. We used the following formula to calculate the sampling weights: sampling weight = proportion of subpopulation in total population/proportion of subpopulation in sample. The subpopulation proportions in rural China’s total population are 37.7% for western China rural communities, 1.4% for resettlement migration communities, 42.0% for central China rural communities, and 18.8% for migrant communities. Next, in our sample, the subpopulation proportions are 82% for western rural communities, 5% for resettlement migration communities, 5% for central rural communities, and 8% for migrant communities. Therefore, the final sampling weights for each subpopulation are 0.46 for western China rural communities (equal to 37.7%/82%), 0.28 for resettlement migration villages (equal to 1.4%/5%), 8.4 for central China rural communities (equal to 42%/5%), and 2.35 for migrant communities (equal to 18.8%/8%). All development scores are nonparametrically standardized for age (measured in months). Controls include child’s age, gender, premature birth, whether the mother is the primary caregiver, maternal age and education, father’s education, whether the mother stays at home, whether the father stays at home, family asset index, and whether the household receives a welfare benefit. We also controlled for Bayley tester fixed effects and county fixed effects. All standard errors account for clustering at the village level. * *p* < 0.1; ** *p* < 0.05; *** *p* < 0.01.

**Table 6:** Correlations between caregiver’s mental health and child development outcomes using ordinary least squares, *n* = 2514.

Variables		Cognitive Scores	Language Scores	Social-Emotional Scores	Motor Scores
		(1)	(2)	(3)	(4)
(1)	Moderate-Depression	0.10	−0.06	−0.16 **	0.00
	(1 = yes)	(0.09)	(0.09)	(0.07)	(0.10)
	Controls	YES	YES	YES	YES
	Tester Fixed Effects	YES	YES	YES	YES
	Adjusted R-squared	0.25	0.27	0.20	0.19
(2)	Moderate-Anxiety	0.00	−0.12 *	−0.20 **	0.01
	(1 = yes)	(0.05)	(0.07)	(0.08)	(0.10)
	Controls	YES	YES	YES	YES
	Tester Fixed Effects	YES	YES	YES	YES
	Adjusted R-squared	0.24	0.27	0.20	0.19
(3)	Moderate-Stress	0.05	−0.05	0.03	0.02
	(1 = yes)	(0.13)	(0.08)	(0.08)	(0.09)
	Controls	YES	YES	YES	YES
	Tester Fixed Effects	YES	YES	YES	YES
	Adjusted R-squared	0.25	0.27	0.20	0.19

Notes: We used sampling weights to calculate the summary statistics for each observation. We used the following formula to calculate the sampling weights: sampling weight = proportion of subpopulation in total population/proportion of subpopulation in sample. The subpopulation proportions in rural China’s total population are 37.7% for western China rural communities, 1.4% for resettlement migration communities, 42.0% for central China rural communities, and 18.8% for migrant communities. Next, in our sample, the subpopulation proportions are 82% for western rural communities, 5% for resettlement migration communities, 5% for central rural communities, and 8% for migrant communities. Therefore, the final sampling weights for each sub-population are 0.46 for western China rural communities (equal to 37.7%/82%), 0.28 for resettlement migration villages (equal to 1.4%/5%), 8.4 for central China rural communities (equal to 42%/5%), and 2.35 for migrant communities (equal to 18.8%/8%). All development scores are nonparametrically standardized for age (measured in months). Controls include child’s age, gender, premature birth, whether the mother is the primary caregiver, maternal age and education, father’s education, whether the mother stays at home, whether the father stays at home, family asset index, and whether the household receives a welfare benefit. We also controlled for Bayley tester fixed effects and county fixed effects. All standard errors account for clustering at the village level. * *p* < 0.1; ** *p* < 0.05.

**Table 7:** Correlations between caregiver’s mental health and positive parenting practices using ordinary least squares, *n* = 2514.

Variables		Told Story Yesterday (1 = Yes)	Read Book Yesterday (1 = Yes)	Sang Song Yesterday (1 = Yes)	Played Game Yesterday (1 = Yes)	Over Two Child Books in Household (1 = Yes)	Times Expressed Affection to Baby (Times)
		(1)	(2)	(3)	(4)	(5)	(6)
(1)	Moderate Depression	−0.04	−0.04	−0.05	−0.07 *	−0.08 ***	−1.48
	(1 = yes)	(0.03)	(0.03)	(0.05)	(0.04)	(0.03)	(1.12)
	Controls	YES	YES	YES	YES	YES	YES
	Tester Fixed Effects	YES	YES	YES	YES	YES	YES
	Adjusted R-squared	0.12	0.14	0.13	0.11	0.16	0.27
(2)	Moderate Anxiety	−0.00	−0.00	0.01	0.00	−0.01	−0.93
	(1 = yes)	(0.03)	(0.04)	(0.05)	(0.04)	(0.06)	(1.51)
	Controls	YES	YES	YES	YES	YES	YES
	Tester Fixed Effects	YES	YES	YES	YES	YES	YES
	Adjusted R-squared	0.12	0.14	0.13	0.11	0.15	0.27
(3)	Moderate Stress	−0.14 ***	−0.05 **	−0.21 ***	−0.12	−0.03	−2.10 *
	(1 = yes)	(0.03)	(0.02)	(0.08)	(0.08)	(0.09)	(1.13)
	Controls	YES	YES	YES	YES	YES	YES
	Tester Fixed Effects	YES	YES	YES	YES	YES	YES
	Adjusted R-squared	0.13	0.14	0.14	0.11	0.15	0.27

Notes: We used sampling weights to calculate the summary statistics for each observation. We used the following formula to calculate the sampling weights: sampling weight = proportion of subpopulation in total population/proportion of subpopulation in sample. The subpopulation proportions in rural China’s total population are 37.7% for western China rural communities, 1.4% for resettlement migration communities, 42.0% for central China rural communities, and 18.8% for migrant communities. Next, in our sample, the subpopulation proportions are 82% for western rural communities, 5% for resettlement migration communities, 5% for central rural communities, and 8% for migrant communities. Therefore, the final sampling weights for each subpopulation are 0.46 for western China rural communities (equal to 37.7%/82%), 0.28 for resettlement migration villages (equal to 1.4%/5%), 8.4 for central China rural communities (equal to 42%/5%), and 2.35 for migrant communities (equal to 18.8%/8%). Controls include child’s age, gender, premature birth, whether the mother is the primary caregiver, maternal age and education, father’s education, whether the mother stays at home, whether the father stays at home, family asset index, and whether the household receives a welfare benefit. We also controlled for Bayley tester fixed effects and county fixed effects. All standard errors account for clustering at the village level. * *p* < 0.1; ** *p* < 0.05; *** *p* < 0.01.

**Table 8:** Correlations between caregiver’s mental health and negative parenting practices using ordinary least squares, *n* = 2514.

Variables		Sometimes Raise Voice or Yell at Baby (1 = Yes)	Sometimes Spank the Baby (1 = Yes)	Sometimes Take Away Toys from Baby (1 = Yes)	Sometimes Do Not Explain Why Baby’s Behavior Is Not Appropriate to Him/Her (1 = Yes)
		(1)	(2)	(3)	(4)
(1)	Moderate Depression	0.08	0.11 *	0.07 **	0.07 *
	(1 = yes)	(0.08)	(0.06)	(0.03)	(0.04)
	Controls	YES	YES	YES	YES
	Tester Fixed Effects	YES	YES	YES	YES
	Adjusted R-squared	0.17	0.13	0.08	0.15
(2)	Moderate Anxiety	0.06 *	0.10 ***	0.07 *	0.02
	(1 = yes)	(0.03)	(0.02)	(0.04)	(0.03)
	Controls	YES	YES	YES	YES
	Tester Fixed Effects	YES	YES	YES	YES
	Adjusted R-squared	0.17	0.13	0.08	0.15
(3)	Moderate Stress	0.02	0.10	0.10	0.04
	(1 = yes)	(0.10)	(0.08)	(0.07)	(0.05)
	Controls	YES	YES	YES	YES
	Tester Fixed Effects	YES	YES	YES	YES
	Adjusted R-squared	0.17	0.13	0.08	0.15

Notes: We used sampling weights to calculate the summary statistics for each observation. We used the following formula to calculate the sampling weights: sampling weight = proportion of subpopulation in total population/proportion of subpopulation in sample. The subpopulation proportions in rural China’s total population are 37.7% for western China rural communities, 1.4% for resettlement migration communities, 42.0% for central China rural communities, and 18.8% for migrant communities. Next, in our sample, the subpopulation proportions are 82% for western rural communities, 5% for resettlement migration communities, 5% for central rural communities, and 8% for migrant communities. Therefore, the final sampling weights for each subpopulation are 0.46 for western China rural communities (equal to 37.7%/82%), 0.28 for resettlement migration villages (equal to 1.4%/5%), 8.4 for central China rural communities (equal to 42%/5%), and 2.35 for migrant communities (equal to 18.8%/8%). Controls include child’s age, gender, premature birth, whether the mother is the primary caregiver, maternal age and education, father’s education, whether the mother stays at home, whether the father stays at home, family asset index, and whether the household receives a welfare benefit. We also controlled for Bayley tester fixed effects and county fixed effects. All standard errors account for clustering at the village level. * *p* < 0.1; ** *p* < 0.05; *** *p* < 0.01.

## References

[1] Goldin C. (2016). Human capital. Handbook of Cliometrics.

[2] Gertler P., Heckman J., Pinto R., Zanolini A., Vermeersch C., Walker S., Chang S.M., Grantham-McGregor S. (2014). Labor market returns to an early childhood stimulation intervention in Jamaica. Science.

[3] Kharas H., Kohli H. (2011). What Is the Middle Income Trap, Why do Countries Fall into It, and How Can It Be Avoided?. Glob. J. Emerg. Mark. Econ..

[4] Schultz T.W. (1961). Investment in Human Capital. Am. Econ. Rev..

[5] Cai F. (2012). Is There a “Middle-income Trap”? Theories, Experiences and Relevance to China. China World Econ..

[6] Zhang L., Yi H., Luo R., Liu C., Rozelle S. (2013). The human capital roots of the middle income trap: The case of China. Agric. Econ..

[7] Khor N., Pang L., Liu C., Chang F., Mo D., Loyalka P., Rozelle S. (2016). China’s Looming Human Capital Crisis: Upper Secondary Educational Attainment Rates and the Middle-income Trap. China Q..

[8] Wang L., Li M., Abbey C., Rozelle S. (2018). Human Capital and the Middle Income Trap: How Many of China’s Youth are Going to High School?. Dev. Econ..

[9] Heckman J.J. (2008). Schools, skills, and synapses. Econ. Inq..

[10] Grantham-McGregor S., Cheung Y.B., Cueto S., Glewwe P., Richter L., Strupp B., The International Child Development Steering Group (2007). Developmental potential in the first 5 years for children in developing countries. Lancet.

[11] Knudsen E.I., Heckman J.J., Cameron J.L., Shonkoff J.P. (2006). Economic, neurobiological, and behavioral perspectives on building America’s future workforce. Proc. Natl. Acad. Sci. USA.

[12] Nelson C.A., Bos K., Gunnar M.R., Sonuga-Barke E.J.S.V. (2011). The neurobiological toll of early human deprivation. Monogr. Soc. Res. Child Dev..

[13] Heckman J.J. (2000). Policies to foster human capital. Res. Econ..

[14] Attanasio O., Cattan S., Fitzsimons E., Meghir C., Rubio-Codina M. (2015). Estimating the Production Function for Human Capital: Results from a Randomized Control Trial in Colombia. Natl. Bur. Econ. Res..

[15] Heckman J.J., Moon S.H., Pinto R., Savelyev P.A., Yavitz A. (2010). The rate of return to the HighScope Perry Preschool Program. J. Public Econ..

[16] Schweinhart L.J., Montie J., Xiang Z., Barnett W.S., Belfield C.R., Nores M. (2006). Lifetime effects: The High/Scope Perry preschool study through age 40. Hum. Resour..

[17] Walker S.P., Wachs T.D., Grantham-Mcgregor S., Black M.M., Nelson C.A., Huffman S.L., Baker-Henningham H., Chang S.M., Hamadani J.D., Lozoff B. (2011). Inequality in early childhood: Risk and protective factors for early child development. Lancet.

[18] Cogill S.R., Caplan H.L., Alexandra H., Robson K.M., Kumar R. (1986). Impact of maternal postnatal depression on cognitive development of young children. Br. Med. J..

[19] Richman N., Stevenson J.E., Graham P.J. (1975). Prevalence of behaviour problems in 3-year-old children: An epidemiological study in London Borough. J. Child Psychol. Psychiatry.

[20] Rutter M., Quinton D. (1984). Parental psychiatric disorder: Effects on children. Psychol. Med..

[21] Rahman A., Patel V., Maselko J., Kirkwood B. (2008). The neglected “m” in MCH programmes—Why mental health of mothers is important for child nutrition. Trop. Med. Int. Heal..

[22] Surkan P.J., Kennedy C.E., Hurley K.M., Black M.M. (2011). Maternal depression and early childhood growth in developing countries: Systematic review and meta-analysis. Bull. World Health Organ..

[23] Galler J.R., Harrison R.H., Ramsey F., Forde V., Butler S.C. (2000). Maternal depressive symptoms affect infant cognitive development in Barbados. J. Child Psychol. Psychiatry.

[24] Whiffen V.E., Gotlib I.H. (1989). Infants of postpartum depressed mothers: Temperament and cognitive status. J. Abnorm. Psychol..

[25] Lovejoy M.C., Graczyk P.A., O’Hare E., Neuman G. (2000). Maternal depression and parenting behavior: A meta-analytic review. Clin. Psychol. Rev..

[26] Downey G., Coyne J.C. (1990). Children of depressed parents: An integrative review. Psychol. Bull..

[27] Yue A., Shi Y., Luo R., Chen J., Garth J., Zhang J., Medina A., Kotb S., Rozelle S. (2017). China’s invisible crisis: Cognitive delays among rural toddlers and the absence of modern parenting. China J..

[28] Luo R., Shi Y., Zhou H., Yue A., Zhang L., Sylvia S., Medina A., Rozelle S. (2015). Micronutrient deficiencies and developmental delays among infants: Evidence from a cross-sectional survey in rural China. BMJ Open.

[29] Xu M., Liu X.H., Du Y.M., Yang Y.H., Li Z.H. (2009). The analysis of infant’s mental and motor development level and influencing factors in the countryside of Shaanxi Province. J. Xi’an Jiaotong Univ..

[30] Yue A., Sylvia S., Bai Y., Shi Y., Luo R., Rozelle S. (2016). The Effect of Maternal Migration on Early Childhood Development in Rural China (Draft December 2016). https://pdfs.semanticscholar.org/034e/d6b5f91ff1564c3c883cec2b956f755dcd19.pdf.

[31] Wang L., Liang W., Yu C., Li M., Zhang S., Sun Y., Ma Q., Bai Y., Abbey C., Luo R. (2018). Are infant/toddler developmental delays a problem across rural China?. Work. Pap..

[32] Albers C.A., Grieve A.J. (2007). Test Review: Bayley, N. (2006). Bayley Scales of Infant and Toddler Development– Third Edition. San Antonio, TX: Harcourt Assessment. J. Psychoeduc. Assess..

[33] Gao L.L., Chan S.W.C., Mao Q. (2009). Depression, perceived stress, and social support among first-time Chinese mothers and fathers in the postpartum period. Res. Nurs. Health.

[34] Wei Q.W., Zhang J.X., Scherpbier R.W., Zhao C.X., Luo S.S., Wang X.L., Guo S.F. (2015). High prevalence of developmental delay among children under three years of age in poverty-stricken areas of China. Public Health.

[35] Phillips M.R., Zhang J., Shi Q., Song Z., Ding Z., Pang S., Li X., Zhang Y., Wang Z. (2009). Prevalence, treatment, and associated disability of mental disorders in four provinces in China during 2001-05: An epidemiological survey. Lancet.

[36] National Bureau of Statistic of the People’s Republic of China (2008). The Second National Agricultural Census of China. http://www.stats.gov.cn/tjsj/pcsj/nypc/nypc2/nm/indexch.htm.

[37] The Central People’s Government of the People’s Republic of China (2006). China Rural Poverty Alleviation and Development Outline. http://www.gov.cn/zwhd/ft2/20061117/content_447141.htm.

[38] National Bureau of Statistic of the People’s Republic of China National Statistic Yearbook 2017. http://www.stats.gov.cn/tjsj/ndsj/2017/indexeh.htm.

[39] National Bureau of Statistic of the People’s Republic of China Monthly Data by province 2018. http://data.stats.gov.cn/easyquery.htm?cn=E0101.

[40] Weiss L.G., Oakland T., Aylward G.P. (2010). Bayley-III Clinical Use and Interpretation.

[41] Azari N., Soleimani F., Vameghi R., Sajedi F., Shahshahani S., Karimi H., Kraskian A., Shahrokhi A., Teymouri R., Gharib M. (2017). A Psychometric Study of the Bayley Scales of Infant and Toddler Development in Persian Language Children. Iran. J. Child Neurol..

[42] Madaschi V., Mecca T.P., Macedo E.C., Paula C.S. (2016). Bayley-III scales of infant and toddler development: Transcultural adaptation and psychometric properties. Paideia.

[43] Yu Y.T., Hsieh W.S., Hsu C.H., Chen L.C., Lee W.T., Chiu N.C., Wu Y.C., Jeng S.F. (2013). A psychometric study of the Bayley Scales of Infant and Toddler Development—3rd Edition for term and preterm Taiwanese infants. Res. Dev. Disabil..

[44] Zakaria S., Seok C.B., Sombuling A., Ahmad M.S., Iqbal S. (2012). Reliability and validity for Malay version of Bayley scales of infant and toddler development—Third edition (Bayley-III): Preliminary study (pp. 116–119). Working paper. Int. Proc. Econ. Dev. Res..

[45] Lowe J.R., Erickson S.J., Schrader R., Duncan A.F. (2012). Comparison of the Bayley II mental developmental index and the Bayley III cognitive scale: Are we measuring the same thing?. Acta Paediatr. Int. J. Paediatr..

[46] Serenius F., Källén K., Blennow M., Ewald U., Fellman V., Holmström G., Lindberg E., Lundqvist P., Maršál K., Norman M. (2013). EXPRESS Group, for the Neurodevelopmental Outcome in Extremely Preterm Infants at 2.5 Years After Active Perinatal Care in Sweden. JAMA.

[47] Bos A.F. (2013). Bayley-Ii Or Bayley-Iii: What Do The Scores Tell Us?. Dev. Med. Child Neurol..

[48] Lovibond S.H., Lovibond P.F. (1995). Manual for the Depression Anxiety Stress Scales.

[49] Henry J.D., Crawford J.R. (2005). The short-form version of the Depression anxiety stress scales (DASS-21): Construct validity and normative data in a large non-clinical sample. Br. J. Clin. Psychol..

[50] Chan R.C.K., Xu T., Huang J., Wang Y., Zhao Q., Shum D.H.K., O’Gorman J., Potangaroa R. (2012). Extending the utility of the Depression Anxiety Stress scale by examining its psychometric properties in Chinese settings. Psychiatry Res..

[51] Wang K., Shi H.S., Geng F.L., Zou L.Q., Tan S.P., Wang Y., Neumann D.L., Shum D.H.K., Chan R.C.K. (2016). Research on translations of tests: Cross-cultural validation of the depression anxiety stress scale-21 in China. Psychol. Assess..

[52] Karrass J., Braungart-Rieker J.M. (2005). Effects of shared parent-infant book reading on early language acquisition. J. Appl. Dev. Psychol..

[53] Luo R., Jia F., Yue A., Zhang L., Lyu Q., Shi Y., Yang M., Alexis M., Sarah K., Rozelle S. (2017). Passive parenting and its association with early child development. Early Child Dev. Care.

[54] Shenfield T., Trehub S.E., Nakata T. (2003). Maternal singing modulates infant arousal. Psychol. Music.

[55] Nelms B.C. (2005). Discipline: Parents need our help. J. Pediatr. Health Care.

[56] Regalado M., Sareen H., Inkelas M., Wissow L.S., Halfon N. (2004). Parents’ discipline of young children: Results from the National Survey of Early Childhood Health. Pediatrics.

[57] Xie S., Wang X., Yao Y. (2006). The application of Bayley Scales of Infant Development in infant nursing. J. Nurs..

[58] Zhou X., Bi B., Zheng L., Li Z., Yang H., Song H., Sun Y. (2014). The prevalence and risk factors for depression symptoms in a rural Chinese sample population. PLoS ONE.

[59] Zhang J., Zhao Z., Zhang G. (2006). Alexithymia of People in Some Rural Community of Shandong Province. Chin. Ment. Health J..

[60] Field T. (1998). Maternal depression effects on infants and early interventions. Prev. Med. (Baltim.).

[61] Lyons-Ruth K., Zoll D., Connell D., Grunebaum H.U. (1986). The depressed mother and her one-year-old infant: Environment, interaction, attachment, and infant development. New Dir. Child Adolesc. Dev..

[62] Murray L. (1992). The Impact of Postnatal Depression on Infant Development. J. Child Psychol. Psychiatry.

[63] Petterson S.M., Albers A.B. (2001). Effects of poverty and maternal depression on early child development. Child Dev..

[64] Aktar E., Majdandžić M., De Vente W., Bögels S.M. (2013). The interplay between expressed parental anxiety and infant behavioural inhibition predicts infant avoidance in a social referencing paradigm. J. Child Psychol. Psychiatry Allied Discip..

[65] O’Connor T.G., Heron J., Golding J., Beveridge M., Glover V. (2002). Maternal antenatal anxiety and children’s behavioural/emotional problems at 4 years. Report from the Avon Longitudinal Study of Parents and Children. Br. J. Psychiatry.

[66] Gordon D., Burge D., Hammen C., Adrian C., Jaenicke C., Hiroto D. (1989). Observations of interactions of depressed women with their children. Am. J. Psychiatry.

[67] Lovejoy M.C. (1991). Maternal depression: Effects on social cognition and behavior in parent-child interactions. J. Abnorm. Child Psychol..

[68] Cohn J.F., Campbell S.B., Matias R., Hopkins J. (1990). Face-to-Face Interactions of Postpartum Depressed and Nondepressed Mother-Infant Pairs at 2 Months. Dev. Psychol..

[69] Goodman S.H., Brumley H.E. (1990). Schizophrenic and Depressed Mothers: Relational Deficits in Parenting. Dev. Psychol..

[70] Livingood A.B., Daen P., Smith B.D. (1983). The depressed mother as a source of stimulation for her infant. J. Clin. Psychol..

